# Early Life Stress Induced by Limited Nesting Material Produces Metabolic Resilience in Response to a High-Fat and High-Sugar Diet in Male Rats

**DOI:** 10.3389/fendo.2015.00138

**Published:** 2015-09-07

**Authors:** Jayanthi Maniam, Christopher P. Antoniadis, Kristy W. Wang, Margaret J. Morris

**Affiliations:** ^1^Department of Pharmacology, School of Medical Sciences, UNSW Australia, Sydney, NSW, Australia

**Keywords:** early life stress, limited nesting material, high-fat and high-sugar diet, insulin and glucose tolerance, liver and plasma triglycerides

## Abstract

Environmental conditions experienced in early life can profoundly influence long-term metabolic health, but the additive impact of poor nutrition is poorly understood. Here, we tested the hypothesis that early life stress (ELS) induced by limited nesting material (LN) combined with high-fat and high-sugar diet (HFHS) post-weaning would worsen diet-related metabolic risk. Sprague-Dawley male rats were exposed to LN, postnatal days 2–9, and at weaning (3 weeks), siblings were given unlimited access to chow or HFHS resulting in (Con-Chow, Con-HFHS, LN-Chow, and LN-HFHS, *n* = 11–15/group). Glucose and insulin tolerance were tested and rats were killed at 13 weeks. LN rats weighed less at weaning but were not different to control at 13 weeks; HFHS diet led to similar increases in body weight. LN-chow rats had improved glucose and insulin tolerance relative to Con-Chow, whereas LN-HFHS improved insulin sensitivity versus Con-HFHS, associated with increased peroxisome proliferator-activated receptor gamma co-activator-1-alpha (*Pgc-1*α) mRNA in muscle. No effect of LN on plasma or liver triglycerides was observed, and hepatic gluconeogenic regulatory genes were unaltered. In summary, this study demonstrates that ELS induced by LN conferred some metabolic protection against insulin and/or glucose intolerance in a diet-dependent manner during adulthood.

## Introduction

Exposure to severe stress during early childhood can have an adverse impact on brain development and mental health outcomes during adulthood (1). The impact of early life stress (ELS) on metabolic disease risk, however, is less well explored. While there is strong evidence in the human linking childhood trauma with increased risk of metabolic diseases, such as insulin resistance, diabetes, and hyperlipidemia (2–4), it is unknown whether these adverse metabolic outcomes are a consequence of lifestyle choices. However, in animal studies, data regarding the impact of ELS on later metabolic health outcomes is controversial (5) with evidence for both improved (6, 7) and worsened (8–11) metabolic disease risk.

Evidence suggests that the modification of disease risk when an individual is exposed to ELS (5) depends on their subsequent lifestyle, but limited work has systematically examined the contribution of these factors to the possible metabolic consequences of ELS (5). There is evidence in support of bad lifestyle choices following ELS, such as increased drug and food addiction (12). For example, adult women who were abused as children were overweight, and this was strongly linked with food addiction (12). On the other hand, given the association between perceived stress with increased intake of high energy, palatable foods (13), the type of diet consumed during adulthood is strongly linked to metabolic profile later in life. There is also some evidence showing that a sub-optimal later environment (sedentary lifestyle, food insecurity, a deficient diet or adult stress exposure) alters the risk for developing metabolic disorders (5). Given the difficulty in assessing the direct contribution of lifestyle and diet to later outcomes in humans, animal models are useful as nutrition and activity can be more readily controlled. Further, use of animal models can provide insight into the underlying mechanisms of any metabolic consequences of ELS.

Poor nutrition, such as consuming a high-fat and high-sugar diet (HFHS), is a known risk factor for metabolic disorders, including hyperglycemia, insulin resistance, hyperlipidemia and increased inflammatory responses. Hence, understanding the consequences of the intake of such food following ELS is critical. Although prevention of ELS is ideal, unfortunately this is not feasible. Hence, it is important to explore other alternatives such as manipulating the adulthood environment (e.g, providing a positive environment) which may minimize metabolic disease risk and provide avenues to identify important targets for intervention. Studies using various models of ELS have examined the impact of later diet and reported different outcomes (5). For example, our lab demonstrated that in response to HFHS diet, male rats exposed to ELS by maternal separation in the first 2 weeks after birth, had marked elevations in plasma insulin, and decreased total white adipose tissue (WAT) mass, independent of body weight compared to HFHS non-stressed controls when measured at 19 weeks of age (10, 11). In support, another model of ELS using maternal deprivation induced hyperinsulinemia and impairments in insulin sensitivity in early adulthood, measured through homeostasis model assessment-estimated insulin resistance (HOMA-IR), in male offspring fed a high-fat diet (HFD) relative to HFD non-deprived controls (14). Recently, a new model of ELS that resembles the human condition of maternal neglect, using limited nesting material (LN), has been established (15). This model has been consistently shown to impair behavior and produce deficits in hypothalamic–pituitary–adrenal axis regulation of offspring; however, no work to date has examined the combined impact of LN and HFHS diet on metabolic function.

Different effects on metabolic profile were observed when rats exposed to ELS consumed a healthy diet post-weaning. For example, when rats exposed to maternal separation were given normal chow, no adverse effect on the metabolic profile was seen (10, 11). Another study showed maternally separated rats had improved insulin sensitivity compared to control rats when maintained on healthy chow (6). In those rats exposed to maternal separation and given a diet deficient in *n*-3 polyunsaturated fatty acid (PUFA), insulin was almost doubled compared to siblings consuming a normal diet (8). Together these studies demonstrate that the type of diet consumed differentially affects metabolic profile during adulthood in those exposed to ELS. While the studies outlined above demonstrate an effect of ELS on insulin and glucose concentrations as a risk marker, they did not comprehensively examine glucose/insulin homeostasis through functional studies, except for one study on primates which demonstrated impaired insulin sensitivity as assessed by glucose clamp (9). Hence, it is not clear if ELS impairs insulin/glucose homeostasis, and how ELS interacts with later poor diet to impact insulin and glucose homeostasis.

In this study, we sought to examine whether a HFHS consumed later in life in those rats exposed to LN adversely affects glucose/insulin homeostasis. Since muscle plays a key role in glucose and insulin uptake and utilization, an array of markers involved in insulin/glucose uptake and utilization were measured, including insulin receptors and glucose transporters. Inflammation, insulin resistance, hepatic glucose production, and altered lipid metabolism are interlinked components of metabolic syndrome (16, 17). Previous work from our lab consistently demonstrated chronic consumption of HFHS diet-induced insulin resistance, lipid accumulation, and increased inflammation (17, 18). Hence, here we also examined whether ELS induced by LN alters the effects of diet on hepatic glucose production and lipid metabolism during adulthood.

With the increase in obesity and the availability of cheap high energy food, it is likely that adverse early exposures and the consumption of such food may co-exist, hence understanding the consequences of choosing a HFHS diet following ELS is essential. To address this important issue, we explored how stress exposure during early development affects later metabolic profile. Using the LN paradigm, we first explored whether ELS produces any metabolic consequences in the face of a healthy diet, examining body composition, glucose/insulin handling, hepatic lipid accumulation, and markers of hepatic glucose metabolism. Second, we sought to determine whether the metabolic complications induced by HFHS diet consumption were altered following LN exposure. Examining the interaction between ELS and diet provides an opportunity to determine whether the type of food consumed may increase vulnerability to, or resilience against diet induced metabolic deficits.

## Materials and Methods

### Animals

All animal procedures were approved by the Animal Care and Ethics Committee of UNSW Australia. Male and Female Sprague-Dawley rats (Animal Resource Centre, Perth, WA, Australia) were maintained in a temperature controlled (21–23^°^C) colony room on a 12-h light/dark cycle (lights on at 0700 h) with *ad libitum* access to standard laboratory chow and water. Mating was carried out in house with one male and four females in a cage. Once pregnancy was confirmed, pregnant dams were housed singly. When a litter was born before 09.00 h, the previous day was designated the day of birth [postnatal day 0 (PND 0)]. Litters comprising 9–15 pups were included and standardized to 12 pups/litter at PND 1 using pups from dams littering on the same day to minimize alterations in maternal behavior and pup nutrition. Litters were housed with the dam in polypropylene cages (20 cm × 32 cm × 19 cm) on wood shavings with a metal lid. On PND 2, two litters were assigned to either normal bedding (control) or LN. The cage environment of LN dams was altered by fitting a metal bottom, raised slightly to allow collection of urine and droppings. Rats in the LN group were provided with a single piece of paper towel as bedding material. Both groups were left undisturbed between PND 2 and 9, after which LN groups received normal bedding material until weaning. Number of male offspring per experimental group was between 11 and 15 depending on the measures.

### Post-weaning diet

At PND 21, pups were weaned and housed 3–4 rats/cage. Male offspring were assigned to either standard laboratory chow (11 kJ/g, energy 12% fat, 21% protein, 65% carbohydrate, Gordon’s Specialty Stockfeeds, NSW, Australia) or standard laboratory chow and palatable HFHS, SF03-020 (20 kJ/g, energy 43% fat, 17% protein, 40% sucrose supplied by Specialty Feeds, Glen Forrest, WA, Australia). Chow, water, and HFHS were available *ad libitum*. This generated four groups: Con-Chow; Con-HFHS; LN-Chow; and LN-HFHS, comprising *n* = 11–15/group. Rats were weighed at weaning and at regular intervals throughout the study. Food and liquid intake over 24 h was recorded at regular intervals by weighing food and bottles before presentation to the rats and again after 24 h. Energy intake per cage was measured and average intake was calculated based on the supplier’s information mentioned above.

### Intraperitoneal glucose tolerance test

Pups underwent an intraperitoneal (i.p.) glucose tolerance test (IPGTT) at 10 weeks of age. Rats were fasted overnight and administered glucose (50%, 2 g/kg i.p., *n* = 8–9/group). For blood collection, a small transverse incision of the lateral tail vein was made using a sterile scalpel. Blood glucose concentrations were measured with a glucometer (Accu-Chek, Roche Diagnostics, Sydney, NSW, Australia) at baseline (0 min) and at 15, 30, 45, 60, 90, and 120 min post-glucose injection. In addition, up to 300 μl of blood was collected at 0, 15, 30, 60, and 120 min post-glucose injection into ethylenediamine tetraacetic acid (EDTA)-coated tubes and maintained at 4°C then centrifuged (9677 × *g*, 23°C, 5 min) to separate plasma for measurement of insulin. The area under the curve (AUC) of the glucose concentrations was calculated for each rat for IPGTT and insulin concentrations during IPGTT.

### Intraperitoneal insulin tolerance test

Pups underwent an i.p. insulin tolerance test at 11 weeks of age. Rats were fasted for 6 h and a baseline glucose level was measured (Accu-Chek). The rats were administered insulin (Actrapid, Novo Nordisk; 100 IU/ml, 1 U/kg i.p., *n* = 8–9/group). Blood samples were obtained as above. Blood glucose concentrations were measured at 15, 30, 45, 90, and 120 min post-injection. The AUC of the glucose concentrations was calculated for each rat.

### Terminal sample collection

At 13 weeks of age, after 16 h fasting, rats were anesthetized by ketamine/xylazine (Ketamine: 100 mg/ml Xylazine: 20 mg/ml, dose: 100/15 mg/kg i.p.). Body weight was measured. Rats were decapitated and trunk blood was collected in heparinized tubes and centrifuged (9677 × g, 23°C, 8 min). The plasma was separated and stored at −20^°^C for subsequent determination of plasma insulin and triglycerides. Liver and left soleus muscle was dissected, weighed and snap frozen in liquid nitrogen and stored at −80°C for subsequent analysis. Left epididymal WAT, retroperitoneal WAT, and visceral WAT were dissected and weighed.

### Hormone and lipid assays

Plasma insulin concentration was measured using a commercial ELISA kit (Millipore, Sydney, NSW, Australia). Prior to liver triacylglycerol analysis, tissue (*n* = 12–15/group) was homogenized using a chloroform–methanol mixture as described elsewhere (19). Plasma and liver triacylglycerol content were determined using a colorimetric assay kit (Roche Diagnostics) with density measured on a BioRad iMark plate reader (BioRad, Sydney, NSW, Australia).

### Quantitative real-time polymerase chain reaction

RNA was extracted using Tri-reagent (Sigma-Aldrich, Sydney, NSW, Australia) and treated with DNase I (Invitrogen, Melbourne, VIC, Australia) as previously described (10, 11) to remove any contaminating genomic DNA and stored at −80^°^C. RNA concentration was determined using a Biospec-nano spectrophotometer (Shimadzu, Sydney, NSW, Australia). Two micrograms of RNA was reverse transcribed to cDNA using Omniscript Reverse Transcription kit (Qiagen, Melbourne, VIC, Australia) and stored at −20^°^C. Quantitative real-time polymerase chain reaction (qRT-PCR) was performed on liver samples using Taqman probes (Applied Biosystems, Melbourne, VIC, Australia) for carbohydrate response element binding protein (*Chrebp*, Rn00580702_m1), glucose-6-phosphatase (*G6pc*, Rn00689876_m1), Glucocorticoid receptor (nuclear receptor subfamily 3, group C, member 1, Rn00565562_m1), hexose-6-phosphate dehydrogenase (*H6pd*, Rn01519771_m1), Fatty acid synthase (*FAS*, Rn01463550_m1), 11-beta hydroxysteroid dehydrogenase 1 (*11*β*HSD-1*, Rn00567167_m1), cytoplasmic phosphoenolpyruvate carboxylase (*Pepck*, Rn01529014_m1), nuclear receptor subfamily 1, group H, member 3 (*Nr1h3/LXR*α, Rn00581185_m1), sterol regulatory element binding transcription factor 1(*Srebp-1c*, Rn01495769_m1), Sirtuin 1 (*Sirt1*, AJD1TRO), Sirtuin 3 (*Sirt3*, Rn01501410_m1), and tribbles homolog 3 (*Trib3*, Rn00595314_m1).

Five housekeepers were assessed and the stability of each was analyzed using Normfinder software. The two housekeepers with the highest stability value, TBP (*TATA box binding protein*, Rn01455646_m1) and beta-actin (Rn00667869_m1), were used as reference genes.

For soleus muscle gene expression related to glucose and insulin homeostasis, we performed qRT-PCR using micro fluid cards (Life Technologies), which were pre-customized with genes of interest including insulin-like growth factor 1 (*Igf1*, Rn99999087), Insulin receptor (*Insr*, Rn00690703_m1), Insulin receptor 1 (*Insr1*, Rn02132493_s1), Insulin receptor 2 (*Insr2*, Rn01482270_s1), Solute carrier family 2 (facilitated glucose transporter) membrane 4 (*GLUT-4*, Rn01471908), sterol regulatory element binding transcription factor 1c (*Srebp-1c*, Rn01495769_m1), uncoupling protein 2 (*UCP2*, Rn01754856_m1) and Peroxisome proliferator-activated receptor alpha (*Pgc-1*α, Rn00580241_m1). We used 1.5 μg of cDNA. One hundred microliters of cDNA/Mastermix (Advanced Fast Mastermix, Life Technologies) mixture was pipetted into the micro fluid cards; after centrifugation, the cards were sealed and qRT-PCR was performed. Housekeepers were assessed as above and the geometric mean of the two housekeepers with the highest stability value, *Ywhaz* (tyrosine 3-monooxygenase/tryptophan 5-monooxygenase activation protein) and *Hprt1* (hypoxanthine phosphoribosyltransferase 1), was used. Analysis was performed using the ΔΔCT method and data expressed relative to a pooled calibrator sample.

### Statistical analysis

Data are presented as mean ± SEM. Bodyweight of pups over time was analyzed using one-way ANOVA with repeated measures, followed by *post hoc* Fisher’s least significant difference (LSD) test. Differences in energy intake, organ weight, plasma hormone concentrations, and mRNA expression in liver and muscle tissues were analyzed using two-way ANOVA followed by *post hoc* Fisher’s LSD. *p* < 0.05 was considered significant.

## Results

### Effects of LN exposure on body weight trajectory

**Figure 1:**
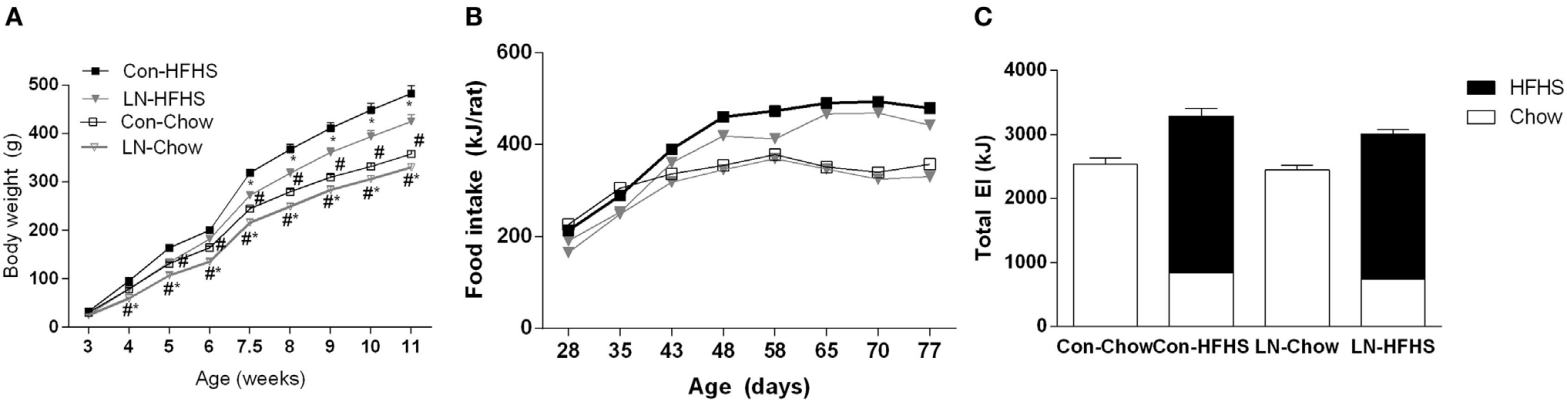
**Body weight trajectory of male pups from weaning to 11 weeks of age for chow (Con-Chow and LN-Chow) and HFHS (Con-HFHS and LN-HFHS) fed groups (A)**. Weekly energy intake (kJ/rat) for Con-Chow, LN-Chow, Con-HFHS and LN-HFHS, *n* = 3–4 cages/group **(B)**. Total weekly energy intake for entire experimental window **(C)**, with energy from chow shown in open and HFHS diet in closed bars. Results are expressed as mean ± SEM, *n* = 11–15/group; data were analyzed by repeated measures one-way ANOVA **(A,B)** and two-way ANOVA **(C)** followed by LSD. Definitions: Con-HFHS [control-HFHS; Con: normal bedding with mother; HFHS: post-weaning, postnatal day (PND) 21, chow, water, + HFHS] and LN-HFHS (LN-HFHS: LN: limited nesting material PND 2–9). ^#^*p* < 0.05 versus rats consuming HFHS (diet effect). *p < 0.05 versus control rats consuming the same diet (LN effect).

Figure 1A demonstrates the body weight trajectory from weaning (3 weeks of age) until 11 weeks in control and LN rats fed chow or HFHS diet. A significant interaction between age and treatment was observed [*F*(24, 440) = 24.71, *p* < 0.0001]. While there were no differences in body weight across groups at weaning, LN male rats fed chow were significantly lighter versus control rats on chow from weeks 4 to 11 (*p* < 0.05, see Figure 1A). In those rats consuming HFHS, LN male rats were significantly lighter compared to control rats consuming the same diet from 7.5 weeks (*p* < 0.05) (see Figure 1A). As expected, HFHS diet consumption significantly increased body weight in control rats (Con-HFHS versus Con-Chow) from 4 weeks of age, while in LN male rats (LN-HFHS versus LN-Chow), an increase in body weight was observed from 5 weeks of age (*p* < 0.05, see Figure 1A).

### Effect of LN exposure on energy intake

When weekly energy intake is considered, there was no significant interaction between age and treatment (LN and HFHS) (*F* < 1, see Figure 1B). When total weekly energy intake was considered (see Figure 1C), there was no significant interaction between LN and HFHS intake (*F* < 1) but a significant effect of HFHS intake was found [*F*(1,11) = 53.49, *p* < 0.0001]. HFHS-fed rats consumed more energy relative to chow-fed rats in LN and control rats (Figure 1C).

### Effects of LN exposure on glucose tolerance

**Figure 2:**
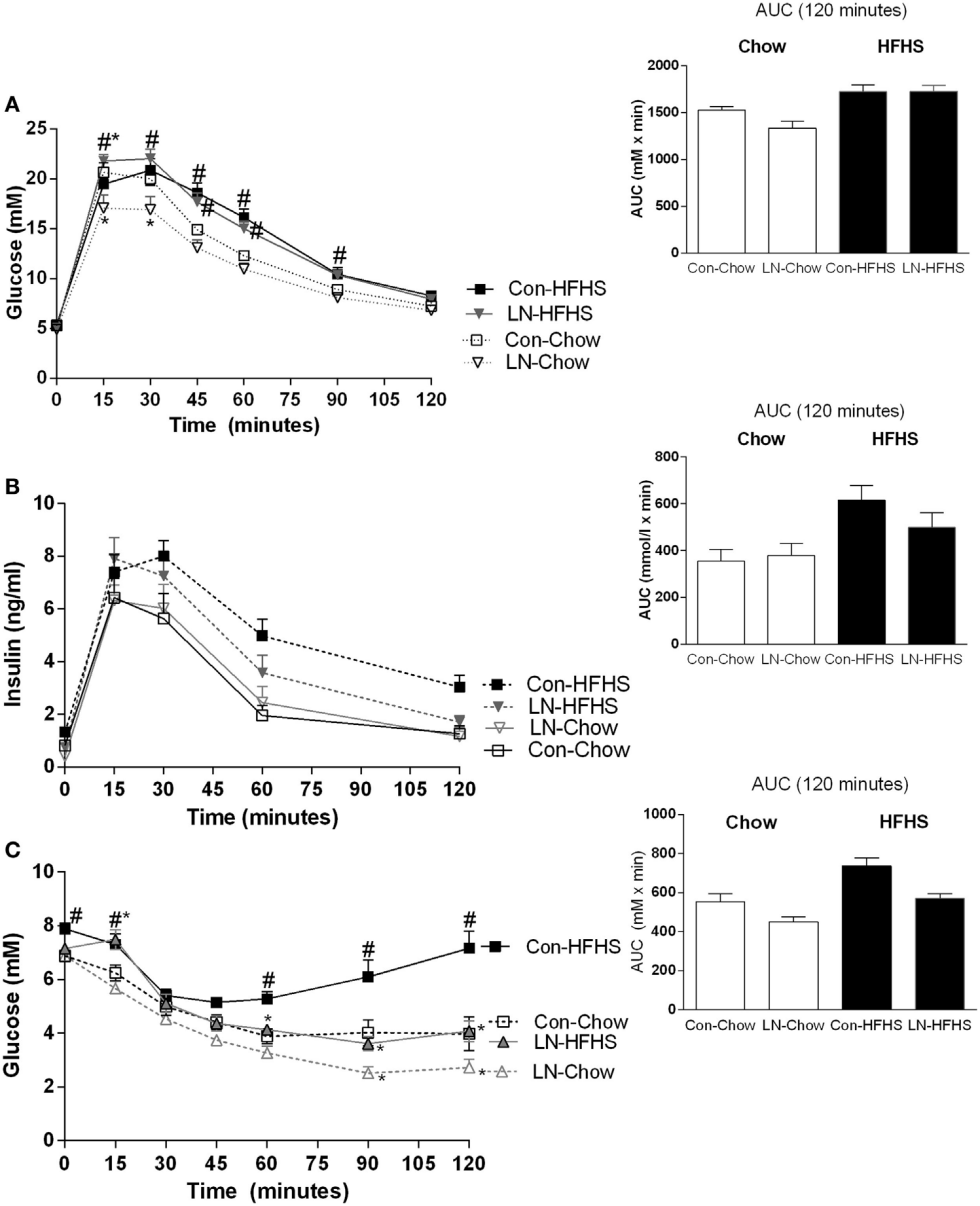
**Glucose tolerance test at 10 weeks of age [(A), 2 g/kg of 50% glucose] and area under curve (AUC) (inset)**. Insulin levels during glucose tolerance test **(B)** and insulin tolerance test **(C)** conducted at 11 weeks. Results are expressed as mean ± SEM *n* = 8–10/group; data were analyzed by repeated measures two-way ANOVA **(A–C)** and two-way ANOVA for AUC [**(A–C)** insets] followed by LSD. Definitions: Con-HFHS [Control-HFHS; Con: normal bedding with mother; HFHS: post-weaning, postnatal day (PND) 21, chow, water, +HFHS] and LN-HFHS (LN-HFHS: LN: limited nesting material PND 2–9). ^#^*p* < 0.05 versus rats consuming chow (diet effect). *p < 0.05 versus control rats consuming chow (LN effect).

When blood glucose concentrations during the GTT test at 10 weeks are considered, a significant interaction between time and treatment [*F*(18, 168) = 4.36, *p* < 0.0001] was observed. In those consuming chow, a lower glucose peak was observed at 15 and 30 min in LN rats relative to control rats (*p* < 0.05, Figure 2A). When AUC was considered, no significant interaction was observed between LN exposure and diet (*p* > 0.05, see Figure 2A inset). Despite no significant difference being detected when considering the AUC during IPGTT across LN and control rats consuming chow (*p* > 0.05, Figure 2A inset), the lower glucose peak exhibited by the LN rats at 15 and 30 min relative to control rats (*p* < 0.05, see Figure 2A) indicates that these rats have improved glucose tolerance in the initial 30 min after a glucose load.

However, in response to HFHS diet, LN-HFHS rats appeared to have similar glucose tolerance as Con-HFHS (see Figure 2A). Con-HFHS rats had higher blood glucose concentrations than their chow counterparts from 45 to 90 min post-glucose injection, while LN-HFHS rats had significantly higher glucose concentrations from 15 to 90 min post-glucose injection (*p* < 0.05, see Figure 2A). When overall AUC for glucose tolerance was considered, there was no significant interaction between LN exposure and HFHS intake (*F* < 1), but as expected, a significant effect of HFHS intake was present [*F*(1,28) = 22.67, *p* < 0.0001] in both LN and control rats (see Figure 2A inset).

### Effects of LN exposure on insulin secretion during IPGTT

Insulin secretion during the GTT was also measured, but there was no significant interaction between time and treatment (*F* < 1, see Figure 2B). When insulin AUC was calculated, there was also no significant interaction between LN exposure and HFHS intake (*F* < 1, Figure 2B inset). However, as expected, a significant main effect of HFHS intake on insulin secretion in response to glucose load [*F*(1,33) = 11.02, *p* < 0.0022] was observed (see Figure 2B inset).

### Effects of LN exposure on insulin tolerance during IPGTT

Insulin tolerance tests were conducted at 11 weeks; in this test, a significant interaction between time and treatment was observed [*F*(18,180) = 6.76, *p* < 0.0001]. In those consuming chow, LN-Chow rats had improved insulin tolerance relative to Con-Chow from 90 min onward (*p* < 0.05, Figure 2C).

However, in those consuming HFHS diet, glucose concentrations post-insulin injection remained markedly higher in the Con-HFHS rats from 60 min post-injection relative to Con-Chow rats (*p* < 0.05, see Figure 2C) but this effect of HFHS intake was absent in LN rats. Interestingly, glucose concentrations in LN-HFHS and LN-Chow rats were similar across all time points post-insulin injection (*p* > 0.05, see Figure 2C). Further, LN-HFHS rats had significantly lower glucose concentrations from 60 min post-insulin injection relative to Con-HFHS rats, which is suggestive of enhanced insulin sensitivity (*p* < 0.01, see Figure 2C).

When overall AUC for insulin tolerance was considered, as expected a significant effect of HFHS intake was observed [*F*(1,30) = 20.50, *p* < 0.0001] in both LN and control rats. In addition, interestingly, LN exposure appeared to improve glucose clearance in response to insulin injection in both chow- and HFHS-fed rats [*F*(1,30) = 16.31, *p* = 0.003, see Figure 2C inset]. However, there was no significant interaction between LN ­exposure and HFHS intake (*F* < 1).

### Effects of LN exposure on offspring body weight, organ mass, and plasma measures

At weaning, body weights were significantly different across groups with reduced body weight in LN rats versus control rats [*F*(3,55) = 15.87, *p* < 0.0001]. At 13 weeks when rats were culled, two-way ANOVA analysis revealed that there was no significant interaction between LN exposure and HFHS intake on body weight (*F* < 1, see Table 1) but there was a significant main effect of LN exposure [*F*(1,53) = 79.26, *p* < 0.0001] and HFHS intake increased body weight [*F*(1,53) = 11.20, *p* = 0.002].

**Table 1:** **Effects of limited nesting material (LN) and high-fat and high-sugar (HFHS) intake on offspring phenotype at 13 weeks**.

	Con	LN	Con-HFHS	LN-HFHS	Significance		
					Interaction	Diet	LN
BW weaning (g)	30.3 ± 0.7	24.7 ± 0.9*	32.7 ± 1.1	26.0 ± 1.1*	–	–	–
BW terminal (g)	357.9 ± 7.8	330.1 ± 8.6	478.1 ± 17.3	425.0 ± 13.9	ns	*p* < 0.0001	*p* = 0.002
Muscle (g)	2.26 ± 0.19	2.06 ± 0.12	3.08 ± 0.06	2.73 ± 0.10	ns	*p* < 0.0001	*p* = 0.041
Muscle (%BW)	0.63 ± 0.04	0.64 ± 0.01	0.65 ± 0.02	0.65 ± 0.02	ns	ns	ns
WAT (g)	3.42 ± 0.28	3.00 ± 0.44	12.43 ± 1.73	9.29 ± 1.03	ns	*p* < 0.0001	*p* = 0.067
WAT (%BW)	0.96 ± 0.07	0.91 ± 0.14	2.52 ± 0.30	2.14 ± 0.19	ns	*p* < 0.0001	ns
Liver (%BW)	3.09 ± 0.09	3.11 ± 0.11	3.35 ± 0.09	3.19 ± 0.07	ns	ns	ns
Liver triglycerides (mg/g)	1.46 ± 0.20	1.23 ± 0.087	5.53 ± 0.79	5.41 ± 0.79	ns	*p* < 0.0001	ns
**Plasma**							
Glucose (mmol/l)	7.59 ± 0.52	6.77 ± 0.48	11.93 ± 0.86^#^	8.22 ± 0.52^*#^	*p* = 0.019	*p* < 0.0001	*p* = 0.02
Insulin (ng/ml)	0.53 ± 0.03	0.46 ± 0.02	0.63 ± 0.06	0.50 ± 0.03	ns	*p* = 0.045	*p* = 0.009
Triglycerides (mg/ml)	0.57 ± 0.07	0.53 ± 0.07	1.58 ± 0.22	1.11 ± 0.18	ns	*p* < 0.001	ns

*Apart from weaning body weight and food intake, the data are terminal, after 16 h fast*.

*Results are expressed as mean ± SEM; data were analyzed by one-way ANOVA for body weight at weaning and two-way ANOVA for other parameters followed by LSD, *n* = 3–4 cages/group for food intake and *n* = 11–15/group for other measures*.

*Abbreviations/definitions: BW, body weight; WAT, total white adipose tissue, includes epididymal, retroperitoneal, and visceral WAT, muscle weight is the sum of soleus and gastrocnemius; Con-HFHS [control-HFHS; Con: normal bedding with mother; HFHS: post-weaning, postnatal day (PND) 21, chow, water + HFHS] and LN-HFHS (LN-HFHS: LN: limited nesting material PND 2–9). Con-HFHS and LN-HFHS groups for body weight at weaning are pre-diet groups. *Post hoc* analysis performed when significant interaction (*p* < 0.05) between diet and LN present*.

*^#^*p* < 0.05 versus rats consuming chow (diet effect)*.

* < 0.05 versus control rats consuming the same diet (LN effect).

For muscle and total WAT mass, LN exposure had no effect on these parameters, independent of the diet consumed as evidenced by no significant interactions between LN exposure and HFHS intake on net mass, or following correction for body weight (*F* < 1). However, as expected HFHS diet increased WAT mass/body weight versus chow-fed rats in both LN and control groups as evidenced by a main effect of diet on WAT mass/body weight [*F*(1,53) = 59.10, *p* < 0.0001, see Table 1]. Whereas for liver, LN exposure or the combination of HFHS intake had no effect on liver mass demonstrated by no significant interaction between LN exposure and HFHS intake, or main effect of LN exposure and HFHS intake on net or body weight adjusted liver mass (*F* < 1) (see Table 1).

Liver triglyceride content was measured as an index of hepatic lipid accumulation. There was no significant interaction between LN exposure and HFHS intake (*F* < 1) on liver triglycerides but as expected a main effect of HFHS intake was present [*F*(1,56) = 67.95, *p* < 0.0001], where HFHS intake increased liver triglyceride four times versus chow counterparts in both LN and control groups (see Table 1). A similar pattern was observed in plasma triglyceride concentrations. HFHS intake increased plasma triglyceride three times in the control rats (Con-HFHS versus Con-Chow) and doubled it in the LN rats (LN-HFHS versus LN-Chow) [*F*(1,41) = 34.46, *p* < 0.0001, see Table 1].

Fasting blood glucose and plasma insulin concentrations were also measured at the end of the study. A significant interaction between LN exposure and HFHS intake was observed in plasma glucose concentrations [*F*(1,51) = 5.911, *p* = 0.019]. There was a main effect of diet [*F*(1,51) = 23.60, *p* < 0.0001] where the intake of HFHS diet significantly increased blood glucose concentrations in control rats by 39% (*p* < 0.0001, see Table 1), but this observation was absent in LN rats (*p* = 0.089, see Table 1). Thus, the glucose concentrations in LN-HFHS were significantly lower compared to Con-HFHS rats (*p* < 0.0001, see Table 1). On the other hand, for fasting insulin concentrations, there was no significant interaction between LN exposure and HFHS intake, but a main effect of HFHS intake [*F*(1,39) = 4.289, *p* = 0.0450] and LN exposure [*F*(1,39) = 7.490, *p* = 0.009] were observed (see Table 1).

### Effects of LN exposure on mediators of insulin/glucose and lipid metabolism in muscle

To examine the effect of LN exposure and HFHS intake on markers of insulin/glucose homeostasis, we measured several key mediators involved in insulin and glucose utilization and transportation in muscle as outlined in Table 2. There were no significant interactions between LN exposure and HFHS intake on all the insulin and glucose metabolism mediators measured, but as expected, there was a main effect of diet on insulin growth factor and insulin receptor mRNA expression in both LN and control groups [*F*(1,28) = 5.216, *p* = 0.03, see Table 2]. Insulin stimulates glucose transport in muscle by provoking the translocation of glucose transporters and glucose enters the muscle cell via facilitated diffusion through the *GLUT-4* transporter, which translocates to the plasma membrane. Here, *GLUT-4* mRNA expression was measured in muscle; there was no significant interaction between LN exposure and HFHS intake, however as expected, a main effect of diet was found (*p* < 0.05, see Table 1). The role of *UCP-2* in muscle is still the subject of debate. However, there is some evidence showing that *UCP-2* is associated with obesity and hyperinsulinaemia. For example, muscle *UCP-2* mRNA expression was reduced in obese subjects (20). *UCP-2* mRNA expression in isolated muscle from the rat has been shown to be increased by insulin treatment *in vitro* (21). Here, LN exposure did not impact muscle *UCP-2* mRNA expression but HFHS intake increased its expression across both groups as evidenced by a main effect of diet [*F*(1,28) = 5.20, *p* = 0.03]. Importantly, *Pgc-1*α, an oxidative metabolism marker that plays a key role in mitochondrial biogenesis and insulin metabolism, was significantly affected by LN exposure and HFHS intake with significant interaction between these two factors [*F*(1,26) = 8.919, *p* = 0.006]. Further, a main effect of HFHS intake [*F*(1,26) = 13.62, *p* = 0.001] and a main effect of LN exposure was found [*F*(1,26) = 4.478, *p* = 0.044]. While HFHS intake had no effect on muscle *Pgc-1*α mRNA expression in control rats, interestingly in LN rats, HFHS intake dramatically increased *Pgc-1*α mRNA expression levels relative to chow counterparts (*p* = 0.0002). Moreover, *Pgc-1*α mRNA expression in LN-HFHS rats was 110% more than the Con-HFHS rats (*p* = 0.0014). Since chronic HFHS intake is a known risk factor for lipid accumulation in muscle, Srebp-1c was measured; however, no significant interaction between LN exposure and HFHS intake was observed. As expected, a significant main effect of diet was observed where increases in *Srebp-1c* were observed in Con-HFHS versus Con-Chow [*F*(1,28) = 4.776, *p* = 0.037, see Table 2], but this appears to be absent in the LN group.

**Table 2:** **Effects of limited nesting material (LN) exposure and high-fat and high-sugar (HFHS) intake on insulin/glucose and lipid metabolism mediators in muscle**.

	Con	LN	Con-HFHS	LN-HFHS	Significance		
					Interaction	Diet	LN
Insulin growth factor	1.11 ± 0.17	1.03 ± 0.16	1.73 ± 0.23	1.93 ± 0.18	ns	*p* < 0.01	ns
Insulin receptor	1.29 ± 0.30	1.29 ± 0.11	1.89 ± 0.28	2.13 ± 0.45	ns	*p* = 0.03	ns
Insulin receptor 1	1.08 ± 0.15	1.22 ± 0.22	1.13 ± 0.17	0.96 ± 0.15	ns	ns	ns
Insulin receptor 2	1.43 ± 0.44	1.20 ± 0.23	2.00 ± 0.38	1.10 ± 0.17	ns	ns	ns
Glucose transporter-4	1.16 ± 0.21	1.28 ± 0.16	1.71 ± 0.16	2.18 ± 0.45	ns	*p* = 0.013	ns
Srebp-1c	1.09 ± 0.17	1.71 ± 0.18	2.11 ± 0.36	1.97 ± 0.35	ns	*p* = 0.04	ns
Pgc-1α	1.12 ± 0.21	0.87 ± 0.17	1.32 ± 0.17	2.78 ± 0.57^*#^	*p* = 0.006	*p* = 0.001	*p* = 0.044
UCP2	1.11 ± 0.17	1.19 ± 0.16	1.81 ± 0.30	1.60 ± 0.28	ns	*p* = 0.03	ns

*Results are expressed as mean ± SEM; data were analyzed by two-way ANOVA followed by LSD, *n* = 6–9/group. The data are terminal, after 16 h fast. *Post hoc* analysis was performed when significant interaction between diet and LN was present. mRNA expression was assessed using microfluid card platform (Life Technologies)*.

*Abbreviations/definitions: Srebp-1c, sterol regulatory element binding transcription factor; Pgc-1α, peroxisome proliferator-activated receptor alpha; UCP2, uncoupling protein 2; Con-HFHS [control-HFHS; Con: normal bedding with mother; HFHS: post-weaning, postnatal day (PND) 21, chow, water, + HFHS] and LN-HFHS (LN-HFHS: LN: limited nesting material PND 2–9)*.

**Post hoc* analysis performed when significant interaction (*p* < 0.05) between diet and LN present*.

*^#^*p* < 0.05 versus rats consuming chow (diet effect)*.

*  0.05 versus control rats consuming the same diet (LN effect).

### Effects of LN exposure on hepatic gluconeogenic and lipid metabolism genes

To examine the effects of LN exposure and HFHS intake on hepatic glucose synthesis, the expression of several key gluconeogenic genes including *Sirtuin 1*, *Sirtuin 3*, *Pepck*, *G6pc*, and *Trib3* were measured as outlined in Table 3. There were no significant interactions between LN exposure and HFHS intake in all the parameters measured. However, as expected a significant HFHS effect was observed in *Pepck* mRNA expression (*p* = 0.001, see Table 3). Interestingly, this is the first time an effect of diet on liver *Trib3* mRNA expression was observed where HFHS intake reduced *Trib3* mRNA expression in both LN and control groups (*p* = 0.02, see Table 3). No effect of LN exposure on *Trib3* mRNA expression was observed.

**Table 3:** **Effects of limited nesting material (LN) exposure and high-fat and high-sugar (HFHS) intake on mediators of hepatic glucose production and lipid metabolism**.

	Con	LN	Con-HFHS	LN-HFHS	Significance		
					Interaction	Diet	LN
**Gluconeogenic Genes**							
Sirtuin 1	1.05 ± 0.08	1.15 ± 0.06	1.04 ± 0.012	0.92 ± 0.06	ns	ns	ns
Sirtuin 3	1.06 ± 0.09	1.02 ± 0.07	0.83 ± 0.07	1.06 ± 0.14	ns	ns	ns
Trib3	1.06 ± 0.09	1.11 ± 0.16	0.80 ± 0.15	0.74 ± 0.11	ns	*p* = 0.02	ns
Pepck	1.06 ± 0.10	1.30 ± 0.11	0.75 ± 0.09	0.80 ± 0.11	ns	*p* = 0.001	ns
G6pc	1.09 ± 0.17	1.35 ± 0.20	1.02 ± 0.16	1.06 ± 0.16	ns	ns	ns
**Lipogenic Genes**							
Srebp-1c	1.11 ± 0.20	0.80 ± 0.11	2.43 ± 0.40	2.55 ± 0.50	ns	*p* < 0.0001	ns
FAS	1.08 ± 0.11	1.33 ± 0.15	0.95 ± 0.13	1.54 ± 0.15	ns	ns	*p* = 0.004
Chrebp	0.99 ± 0.07	1.10 ± 0.03	0.96 ± 0.07	1.27 ± 0.09	ns	ns	ns
**Glucocorticoid Metabolism Genes**							
11βHSD-1	1.02 ± 0.06	1.06 ± 0.07	0.64 ± 0.07	0.56 ± 0.05	ns	<0.0001	ns
GR	0.99 ± 0.07	0.98 ± 0.05	1.23 ± 0.10	1.19 ± 0.07	ns	*p* = 0.028	ns
Lxr-α	1.03 ± 0.07	0.96 ± 0.07	0.86 ± 0.07	0.94 ± 0.07	ns	ns	ns
H6PD	1.06 ± 0.09	1.03 ± 0.08	0.83 ± 0.06	1.08 ± 0.14	ns	ns	ns

*Results are expressed as mean ± SEM; data were analyzed by two-way ANOVA followed by LSD, n = 11–15/group. mRNA expression was assessed using regular RT-PCR using Taqman pre-optimized primers. The data are terminal, after 16 h fast*.

*Abbreviations/definitions: Con-HFHS [control-HFHS; Con: normal bedding with mother; HFHS: post-weaning, postnatal day (PND) 21, chow, water, + HFHS] and LN-HFHS (LN-HFHS: LN: limited nesting material PND 2-9). H6PD, hexose-6-phosphate dehydrogenase; Trib3, tribbles homolog 3; Pepck, cytoplasmic phosphoenolpyruvate carboxylase; Chrebp, carbohydrate response element binding protein, G6pc, glucose-6-phosphatase; Srebp-1c, sterol regulatory element binding transcription factor; FAS, fatty acid synthase; LXRα, nuclear receptor subfamily 1, group H, member 3; 11βHSD-1, 11-beta hydroxysteroid dehydrogenase 1; GR, nuclear receptor subfamily 3, group C, member 1*.

Given tissue glucocorticoid metabolism mediators, including (*11*β*HSD-1* and glucocorticoid receptor), also regulate gluconeogenic enzymes, including *Pepck* and *G6Pc*, the gene expression of *11*β*HSD-1*, and glucocorticoid receptor were measured. We observed no significant interaction between LN exposure and HFHS intake on *11*β*HSD-1* and glucocorticoid receptor mRNA expression (*F* < 1); however, a main effect of HFHS diet was observed in *11*β*HSD-1* [*F*(1,41) = 43.28, *p* < 0.0001] and glucocorticoid receptor [*F*(1,46) = 5.148, *p* = 0.028] mRNA expression.

We also measured *FAS* and *Srebp-1c* mRNA, which regulates genes related to lipid synthesis, and as expected, a significant diet effect was observed in *Srebp-1c* mRNA expression in the liver across both LN and control groups (*F*(1,54) = 22.32, *p* < 0.0001, see Table 3) with no significant interaction between LN exposure and HFHS intake (see Table 3). For *FAS* expression, while there was no significant interaction between LN exposure and diet, interestingly, a main effect of LN exposure [*F*(1,48) = 9.116, *p* = 0.0041] was observed, where LN exposure appeared to increase *FAS* mRNA expression in HFHS relative to Con-HFHS rats. *Chrebp* induces lipogenic gene expression, and here, *Chrebp* mRNA expression was not affected by LN exposure or HFHS diet (*F* < 1).

## Discussion

This study expands the understanding of the impact of chronic consumption of HFHS from weaning on glucose and insulin homeostasis, lipid accumulation, and associated mediators of these systems in muscle and liver in male rats exposed to ELS during the first week of life. LN exposure differentially affected glucose and insulin tolerance during adulthood, which was dependent on the diet the animals consumed. Specifically, LN-chow rats had improved glucose and insulin tolerance relative to control rats given chow and LN-HFHS rats were glucose intolerant, but exhibited improved insulin sensitivity versus Con-HFHS rats. Moreover, LN-HFHS rats had lower fasting glucose versus Con-HFHS rats. Other metabolic parameters, including liver triglycerides and their associated gene expression in the liver, were not affected by LN exposure in both diets. Hepatic gluconeogenic regulatory genes were also not altered.

### LN rats were lighter at weaning but experienced “catch up” growth in chow- and HFHS-fed groups

In our hands, ELS induced by LN resulted in lighter rats relative to control unstressed rats at weaning; however, these rats appeared to experience catch-up growth where LN rats had similar body weight as the control rats consuming the same diet at 13 weeks of age. This was in line with similar energy intake across both the LN and Con groups. Another study using the LN model also reported catch-up growth as rats matured (22). However, pups exposed to another model of ELS, maternal separation, showed no effect on body weight compared to control rats consuming regular diet at weaning (10). The LN model results in pups receiving fragmented care (15) versus maternally separated pups receiving enhanced arched back nursing (10). The differences in care provided by the dams across these two models may explain the differential impact of the ELS model on body weight at weaning. In addition, the fact that LN pups weighed less than the control rats at weaning likely suggests that these LN rats were also nutritionally challenged due to the nature of care provided by LN dams to their pups.

### LN exposure modified glucose and insulin tolerance in a diet-dependent manner

In terms of glucose and insulin handling, LN exposure differentially affected glucose and insulin tolerance in a diet-dependent manner. In those consuming chow, improved glucose clearance in the initial 30 min of GTT in LN rats versus control rats seems to be linked to enhanced insulin sensitivity as demonstrated by insulin tolerance test data. This data highlights that LN exposure produced some beneficial effects in maintaining glucose homeostasis in response to a regular diet. However, another study in primates using variable foraging demand as a model of ELS demonstrated that offspring that went on to consume chow had developed insulin resistance at 4 months of age, as measured by insulin clamps (9). The reasons for the differences in observations are likely related to differences in species, experimental model, and even the age of assessment. Whether the improved glucose tolerance and insulin sensitivity observed in LN-chow rats remains as these animals age is unknown.

We have consistently showed that chronic consumption of HFHS impaired glucose and insulin tolerance (23, 24). Here, glucose tolerance in response to HFHS diet was similar between LN and Con rats and, as expected, HFHS intake delayed glucose clearance in control rats relative to their chow counterparts. However, it is important to highlight that while the HFHS diet effects on glucose tolerance in Con rats appeared from 45 min post-glucose injection, for LN rats, the diet effect was apparently earlier at 15 min post-glucose injection. This suggests that LN rats consuming HFHS diet responded differently to a glucose challenge versus unstressed Con rats. Despite being glucose intolerant, LN-HFHS rats failed to show impaired insulin tolerance as observed in Con-HFHS rats. In addition, in the face of HFHS diet, LN rats exhibited enhanced insulin sensitivity relative to controls. The lower insulin response to glucose challenge seen during the second phase of GTT, albeit statistically non-significant, was also in line with the improved insulin sensitivity observed in LN-HFHS versus Con-HFHS rats.

These findings are the first demonstration showing ELS in the form of LN enhanced insulin sensitivity in response to HFHS intake. One of the few studies performed examined the impact of HFHS intake on fasting insulin concentrations and insulin sensitivity index following maternal separation – and no effect of HFHS diet was observed in maternally separated rats versus control rats fed HFHS diet (6). Taken together, these data further suggest that ELS provides some protection against adverse effects induced by HFHS on insulin homeostasis. The underlying mechanism as to why LN exposure improved insulin sensitivity and lowered insulin response in response to HFHS is unclear. There are other physiological stressors, such as dietary restriction, that are known to improve insulin sensitivity across many species (25, 26). The fact that LN rats were lighter at weaning means that this model had some features that coincided with mild dietary restriction. In models of dietary restriction, improvement in mitochondrial biogenesis was demonstrated through increases in muscle *Pgc-1*α (27) and *UCP2* (28) mRNA expression, and this mechanism was proposed to play a role in the improved insulin sensitivity (29, 30). The likely contribution of changes in the expression of these genes will be discussed below.

It is also important to note that in our hands, while changes in glucose and insulin tolerance were apparent, the overall AUC for these tests was not significantly altered. Thus, future work should use a more robust test for insulin sensitivity, such as an insulin clamp, to validate the differential effects of diet on insulin sensitivity following LN exposure observed in this study. The mechanism(s) underlying this altered response to diet in LN rats are unclear. To understand the potential mechanisms mediating the effects of LN on insulin/glucose homeostasis, several key mediators of insulin and glucose metabolism were measured in muscle. As expected, we observed a diet effect on insulin growth factor, insulin receptor, and *GLUT-4*, but LN exposure did not differentially affect these markers. In muscle cells, elevated levels of *Pgc-1*α stimulated insulin-sensitizing effects via the upregulation of selected genes involved in fatty acid β-oxidation, glucose transport, and oxidative phosphorylation (31, 32). Further, increases in muscle *Pgc-1*α mRNA has recently been shown to be associated with increased insulin sensitivity in both animals and humans (33, 34). In addition, skeletal muscle specific *Pgc-1*α KO mice exhibited glucose intolerance pointing to the importance of *Pgc-1*α in glucose regulation in skeletal muscle (25). The upregulation of *Pgc-1*α mRNA expression we observed in LN-HFHS rats relative to Con-HFHS rats may be associated with the enhanced insulin sensitivity observed in LN-HFHS rats; however, the direct contribution of *Pgc-1*α in the improvement of insulin sensitivity needs to be systematically validated in future work using knock-out models.

### LN exposure had no effects on plasma and hepatic lipid profile

In this study, even though our findings were consistent with previous work showing HFHS intake significantly increased plasma and liver triglyceride concentrations, contrary to our hypothesis, LN exposure did not modify lipid levels in chow- or HFHS-fed rats. To the best of our knowledge, there is no evidence, thus far, on the impact of ELS on hepatic lipid accumulation in either human or animal studies. It was interesting to note that despite no significant interaction between LN exposure and diet, a main effect of LN exposure on lipogenic gene (*FAS*) expression in the HFHS-fed rats where LN rats given HFHS diet had increased *FAS* mRNA expression. Hence, we examined if LN exposure modifies expression of *Chrebp* and *Srebp-1c* mRNA, which regulates the induction of lipogenic genes, such as *FAS*, in response to glucose and insulin, respectively (35). In this study, hepatic *Chrebp* and *Srebp-1c* were not affected by LN exposure in either chow- or HFHS-fed rats. We also examined liver histology for any signs of lipid accumulation following LN exposure in HFHS-fed rats. Histological investigation of the liver revealed a greater accumulation of lipid vacuoles within hepatocytes in those consuming HFHS across LN and control rats. However, LN exposure had no impact on lipid accumulation in rats given chow or HFHS diet (data not shown).This study provides the first evidence that ELS in the form of LN produced no additive effect on the HFHS diet induced increases in plasma and hepatic lipid accumulation.

### LN exposure had no effects on hepatic glucose gene expression

In response to physiological challenges, such as fasting, the liver maintains glucose homeostasis. Hence, long-term fasting initiates gluconeogenesis (36) by increasing the expression of key gluconeogenic enzymes *Pepck* and *G6pc* (37). Here, we observed no differences in the expression of *Pepck* and *G6pc* mRNA in the liver across LN and control rats when rats were fasted for 16 h. However, we found *Pepck* mRNA was reduced by HFHS diet. Hepatic *Trib3* expression is reported to be highly expressed in animal models of diabetes (38), hence promoting hyperglycemia presumably by increasing glucose production in the liver. *Trib3* mRNA expression in liver biopsy samples of obese subjects was strongly correlated with *Pepck* mRNA (39). However, here, HFHS intake halved the expression of *Trib3* mRNA in LN and control rats as evidenced by a main effect of diet and this was in line with reduction of *Pepck* mRNA by the diet.

Hepatic *11*β*HSD-1* also plays a role in regulating key hepatic gluconeogenic enzymes, including *Pepck* and *G6Pc* through the amplification of glucocorticoid receptor-mediated tissue glucocorticoid action, and is crucially dependent on H6PD to generate NADPH (40, 41). Increased activity of *11*β*HSD-1* was observed in non-fasted rats versus fasted rats (40) and in the same study, *11*β*HSD-1* activity, H6PD, and glucocorticoid receptor mRNA expression were increased by glucose in a dose-dependent manner (40). However, here, under conditions of 16 h fasting, HFHS intake almost halved *11*β*HSD-1* mRNA in the liver across both LN and control rats and increased glucocorticoid receptor mRNA expression. These observations are in line with no changes in the expression of glucose synthesis genes (*Pepck* and *G6pc*) after 16 h. Further, this study has produced the first evidence that hepatic gluconeogenic gene expression is not influenced by LN exposure in chow- and HFHS-fed rats.

## Conclusion

Overall, this study demonstrates that ELS induced by LN conferred metabolic protection against insulin and/or glucose tolerance during adulthood. Particularly in those consuming chow and previously exposed to LN, signs of improved glucose clearance were observed in the first 30 min post-glucose challenge and enhanced insulin sensitivity in the last 30 min following insulin challenge. In those consuming a HFHS, LN did not impact glucose tolerance but modestly enhanced insulin sensitivity. This was associated with increased *Pgc-1*α expression in the muscle. We hypothesize that the altered response to LN exposure according to the diet consumed is related to developmental programing of glucose/insulin metabolism in peripheral tissues that enabled LN rats to demonstrate enhanced insulin sensitivity even when challenged with HFHS. However, whether this metabolic profile can be maintained as these animals age is yet to be explored. Further, LN exposure produced no effects on lipid accumulation in plasma or liver, and the relevant lipid markers were also not altered, regardless of diet. Moreover, hepatic glucose metabolism genes were also not altered by LN exposure in both diets. Overall, the results support the notion of greater metabolic “resilience” in LN rats, as previously discussed in Maniam et al. 2014 (5).

## Author Contributions

Designed the experiments: JM and MM. Performed the experiments: JM, CA, KW, and MM. Analyzed the data: JM, CA, KW, and MM. Wrote the paper: JM, CA, KW, and MM.

## Conflict of Interest Statement

The authors declare that the research was conducted in the absence of any commercial or financial relationships that could be construed as a potential conflict of interest.
